# Predictors of migration in an HIV hyper-endemic rural South African community: evidence from a population-based cohort (2005–2017)

**DOI:** 10.1186/s12889-022-13526-w

**Published:** 2022-06-07

**Authors:** Armstrong Dzomba, Hae-Young Kim, Andrew Tomita, Alain Vandormael, Kaymarlin Govender, Frank Tanser

**Affiliations:** 1 Discipline of Public Health Medicine, Africa Health Research Institute(AHRI), University of KwaZulu-Natal, KwaZulu-Natal Province, K-RITH Tower Building, 719 Umbilo Road, Private Bag X7, Congella, Durban, South Africa; 2 KwaZulu-Natal Research Innovation and Sequencing Platform (KRISP), University of KwaZulu-Natal, Durban, South Africa; 3 Medical Research Council (MRC)/Wits Rural Public Health and Health Transitions Research Unit (Agincourt), University of the Witwatersrand, Acornhoek, South Africa; 4 Department of Population Health, New York University Grossman School of Medicine, New York City, NY USA; 5 Centre for Rural Health, School of Nursing and Public Health, University of KwaZulu-Natal, Durban, South Africa; 6 Heidelberg Institute of Global Health (HIGH), University of Heidelberg, Heidelberg, Germany; 7 Department of Medicine, Stanford University, Stanford, USA; 8 Health Economics and HIV and AIDS Research Division (HEARD), University of KwaZulu-Natal, Durban, South Africa; 9 Centre for the AIDS Programme of Research in South Africa (CAPRISA), University of KwaZulu-Natal, Durban, South Africa; 10 Lincoln Institute for Health, University of Lincoln, Lincoln, LN6 7TS UK

**Keywords:** Migration, Migration incidence, Transients and Migrants, Antiretroviral Therapy, Human Immunodeficiency Virus

## Abstract

Globally, South Africa hosts the highest number of people living with HIV (PLHIV) and the unique legacy of internal labour migration continues to be a major driver of the regional epidemic, interrupting treatment-as-prevention efforts. The study examined levels, trends, and predictors of migration in rural KwaZulu-Natal Province, South Africa, using population-based surveillance data from 2005 through 2017. We followed 69 604 adult participants aged 15–49 years and recorded their migration events (i.e., out-migration from the surveillance area) in 423 038 person-years over 525 397 observations. Multiple failure Cox-regression models were used to measure the risk of migration by socio-demographic factors: age, sex, educational status, marital status, HIV, and community antiretroviral therapy (ART) coverage. Overall, 69% of the population cohort experienced at least one migration event during the follow-up period. The average incidence rate of migration was 9.96 events and 13.23 events per 100 person-years in women and men, respectively. Migration rates declined from 2005 to 2008 then peaked in 2012 for both women and men. Adjusting for other covariates, the risk of migration was 3.4-times higher among young women aged 20–24 years compared to those aged ≥ 40 years (adjusted Hazard Ratio [aHR] = 3.37, 95% Confidence Interval [CI]: 3:19–3.57), and 2.9-times higher among young men aged 20–24 years compared to those aged ≥ 40 years (aHR = 2.86, 95% CI:2.69–3.04). There was a 9% and 27% decrease in risk of migration among both women (aHR = 0.91, 95% CI: 0.83 – 0.99) and men (aHR = 0.73, 95% CI 0.66 – 0.82) respectively per every 1% increase in community ART coverage. Young unmarried women including those living with HIV, migrated at a magnitude similar to that of their male counterparts, and lowered as ART coverage increased over time, reflecting the role of improved HIV services across space in reducing out-migration. A deeper understanding of the characteristics of a migrating population provides critical information towards identifying and addressing gaps in the HIV prevention and care continuum in an era of high mobility.

**Supplementary Information:**

The online version contains supplementary material available at 10.1186/s12889-022-13526-w.

## Introduction

South Africa has the highest number of people living with HIV (7.2 million) and runs the world’s largest HIV treatment programme (4.4 million) [1]. Over the last decade, the rapid scale-up and increasingly wide coverage of antiretroviral therapy (ART) led to large declines in AIDS-related deaths and new infections [2, 3], with the latter being almost halved between 2011–2016 [4, 5]. However, HIV incidence rates continue to remain high, with an estimated 240 000 adults newly infected in 2019 alone [1]. Widespread internal mobility and frequent geographical relocations have been strongly linked with increased HIV transmission, risky sexual behavior and interruption of care continua among people living with HIV (PLHIV) [6–26], sustaining the epidemic. South Africa has exceptionally high internal migration rates compared to other countries in sub-Saharan Africa (SSA) [23], having significantly increased since the advent of democracy. Newer forms of movement such as frequent short-distance migration largely involving women exist parallel to historically male-dominated long distance labour mobility, altering profiles of migrants [19, 25]. In rural KwaZulu-Natal Province communities, high out-migration flows to urban locations have been observed [27] and linked to better amenities and employment opportunities in larger cities [27]. As such, a previous study reported that ~ 64% of the adult resident population had migrated at least once between 2005 and 2011 [28], demonstrating the persistence of movement outside the rural households.

The current HIV treatment and prevention landscape builds on existing evidence that population-level reductions in the transmission of HIV could be achieved by leveraging expanded coverage of services [1]. The 95–95-95 targets aimed to have 95% of people living with HIV (PLHIV) know their status, of whom 95% can access ART, and of whom 95% are virally suppressed by 2030 as an indication of their successful engagement in care [1]. Universal voluntary HIV counselling and testing, accompanied by immediate initiation of antiretroviral therapy (ART) for all those diagnosed HIV-infected, as well as Universal Test and Treat (UTT), has been the principal strategies through which the 95–95-95 targets are to be achieved. Despite being on track to achieve the first and last 95 in South Africa, only approximately 70% of PLHIV were receiving ART in 2020, with substantial variations by age and sex among those virally suppressed [29]. Moreover, efforts to increase ART coverage have been a challenge for migrant populations to be retained in the HIV care continuum [9]. Available evidence has a demography focus and predate the universal HIV treatment dispensation [27], thus examining trends, incidence and predictors of mobility is both timely and an essential first step towards identifying impactful migrant-sensitive HIV prevention and care interventions.

Motivated by lack of evidence, there is need to deepen our understanding of the mobility patterns and potential drivers of migration for PLHIV in light of large-scale treatment-as-prevention HIV interventions, such as the UTT currently underway in South Africa. Unfortunately, most HIV interventions fail to appropriately account for the spatial and temporal nature of mobility [30, 31], given that migration intrinsically affects exposure time to HIV services within geographies. For this reason, it is crucial to fill this gap in knowledge and investigate the socio-demographic, clinical and community predictors of migration for intervention target/profile setting, as highly mobile individuals are well known to encounter test/treatment access and retention challenges that can potentially jeopardize the chance of ending the epidemic and preventing further HIV drug resistance [23, 31] in South Africa. In this study, we quantified the role of socio-demographic, clinical and community predictors against out-migration incidence using one of Africa’s largest population-based cohorts in an HIV hyper-endemic rural community in South Africa among ~ 70,000 individuals, both HIV-positive and negative. In particular, we focused on age, sex, educational status, marital status, HIV and community antiretroviral therapy (ART) coverage, and measured the incidence rates per year.

## Methods

### Study design

We analyzed data from the population-based longitudinal surveillance system conducted by the Africa Health Research Institute (AHRI), which is located in the rural uMkhanyakude District of northern KwaZulu-Natal Province. The surveillance includes members of all households located in the 438 km^2^ surveillance area, with a population of approximately 100 000 resident and non-resident members from 11 000 households [3]. AHRI has collected comprehensive socio-demographic information since 2000 on household residencies, mobility patterns and migration, while HIV survey data are available from 2004. ART information is regularly updated on patient records in local primary health care (PHC) clinics and retrievable from the electronic patient management system (Tier.Net) database. Surveys capturing demographic events that can change the structure of the household, such as births, deaths and migrations from key household informants every 4–6 months, were conducted by trained fieldworkers. Data on other socio-economic, health exposures and outcomes, such as education and HIV status, were collected annually since 2004, while ART information was captured from local clinics’ HIV treatment and care program, as highlighted above. Household and individual surveys are linked longitudinally to each other and over time through unique individual and household identification. For HIV surveillance, eligible participants aged 15-years and older are interviewed in private by the same fieldworkers, who also extract blood from consenting participants by finger-prick for annual HIV testing.

This community is characterized by frequent migration (32% of women and 38% of men were non-resident in 2008), and high levels of residential instability, with 33% of those regarded as household members in the surveillance area not residing within the registered households [28]. Local employment is scarce, and residents often migrate for work outside the area. Levels of mobility in the region have risen in recent decades, aligned with rapid socio-economic transformations and the increased labour participation of women. For example, economically active adult women increased from 31 to 41% between 2008 and 2011 in those of working age [32]. Circular migration predominates in this setting, with individuals migrating repeatedly between rural areas, semi-urban towns and the rural perimeters of cities. Women are somewhat more likely than men to undertake any form of migration, although sex differentials in migration trends differ by migration distances [27].

Other characteristics of the surveillance area include low marital rates (i.e. only 20% of conjugal couples among those 20–29 years were married), late marriages, high partnership dissolution rates among older women and younger men [33–35], multiple sexual partnerships as well as by poor knowledge and disclosure of HIV status [33]. KwaZulu-Natal Province bears the largest HIV burden in South Africa, with adult HIV prevalence in this region being 36% in 2017 [36] and disproportionate provision of HIV services particularly in poorer settings [37–39]. ART coverage increased rapidly since 2004, primarily via nurse-led public sector ART programs. The expanded scale-up of ART increased its coverage from 2.1% in 2005 to 24.6% in 2010, and to 50.6% in 2017 among HIV-positive women, and from 1.5% in 2005 to 21.4% in 2010 and 38.4% in 2017 among HIV-positive men [4].

### Patient and public involvement

AHRI has been working with the communities of the 11 000 households of the study site since 2000, with close consultation of the population as well as their political and traditional hierarchies. All study consecutive waves are discussed with the Community Advisory Board (CAB) and representatives of all villages before each census launch. Data collection (i.e., including HIV testing in 2003 and beyond) was based on existing knowledge of the HIV burden in the general population of this community, and discussions with the CAB serving as an input to the study design. The recruitment of participants was conducted after community engagement, i.e., informing communities (a meeting in each village) about the study. Finally, after each census is completed, the key findings are communicated back to the community through routine meetings with representatives of local authorities and Community Roadshows. However, for this study, neither patients nor the public were not involved the design, or conduct, or reporting, or dissemination plans of the research. All methods were carried out in accordance with relevant guidelines and regulations and informed consent was obtained from all the study participants.

### Ethics statement

The Biomedical Research Ethics Committee of the University of KwaZulu-Natal (BREC) Durban, South Africa, gave full ethics approval for this study Ref. no. BE283/17.

### Migration events

The primary outcome of the study was the outgoing external migration among women and men aged 12 years or older. Our study comprised of individuals who were residents when first observed and had previously moved out and back into the study area into the AHRI setting between 2004 and 2017. We defined migration as a movement out of the surveillance area (i.e. out-migration), and categorised a migrant as a person who had experienced an out-migration event from the study area and a non-migrant as having no migration history. Fieldworkers routinely collect migration history data for migrants and non-migrants from key household informants, including details such as place of destination and date on which every migration event occurred. ﻿These migration events lasted varying periods of time over the course of observation. An explicit focus on out-migration enabled us to examine the pre-migration characteristics of mobile adults and assess one important end (origin) of the migration axis in a rural community with high cyclical livelihood migration to urban destinations.

### Predictors

With the exception of sex, time-varying risk factors included: age (15–19; 20–24; 25–29; 30–34; 35–39; ≥ 40 years); educational attainment (no schooling; primary, secondary, tertiary, unknown educations status); marital status (single, married (monogamous/polygamous), separated or divorced, or unknown); HIV status (HIV negative and positive), antiretroviral therapy (a continuous variable) and percentage of community coverage. ART coverage, defined as the proportion of individuals HIV positive receiving ART for each community within the AHRI study site as previously described [3].

The marital status and educational attainment variables had missing data for 15% and 18.7% of the records, respectively. Response rates for HIV were low and prone to missing data, and between 2005 and 2017, 48.7% of total records had no data on HIV status. Evidence from the study area found substantially high participation rates over time but lower over single wave surveys, which is an expected outcome [37, 38]. To help address missingness of data, we categorized values as missing per each variable, thus retaining data for all individuals across different models.

### Statistical analysis

First, we determined the prevalence of residency and non-residency among both women and men between 2005 and 2017 by calculating mid-year frequencies and percentages for residency status by sex (i.e. period of time during which an individual was either present or absent in the surveillance area). Secondly, we estimated migration incidence rates per each year and socio-demographic and health/clinical factors. Thirdly, we fitted Anderson-Gill models to estimate hazard ratios for the association between the risk factors (age, sex, education status, marital status, HIV status, community ART coverage) and out-migration events. We repeated the same analysis to generate hazard ratios stratified by sex, as past studies have shown that male and female have different motivations to of migrate [19, 20]. All models (univariate and multivariate) were fitted employing a counting notation as described by Lin and Zelterman [38]. To account for clustering of observations within each individual, and to control for follow-up time of individuals, we estimated robust standard errors in the analysis. The cox models fitted a unique baseline hazard function per each successive migration event. In addition, we performed sensitivity analyses (i.e. complete case analysis and repeating the statistical stratified by HIV status categories) to diagnose and adjust for selection effects, which are described in the results section. Analyses were performed using Stata 14.0 software (StataCorp, College Station, Texas, USA).

## Results

### Residency

Table 1 presents the data for the resident and non-resident adult study participants between 2005 and 2017. For each year, more than 24% of both women and men were non-residents in the surveillance area. The proportion of non-residents was lower in women than in men, although non-residence similarly increased from 24 to 33% in women and 29% to 40% in men over time.

**Table 1:** Mid-year estimates, residency status by sex and exposure year 2005–2017

Year	Women				Men			
	Non-residents		Residents		Non-residents		Residents	
	N	%	N	%	N	%	N	%
2005	13 887	25.63%	40 304	74.37%	15 749	31.97%	33 514	68.03%
2006	14 339	26.09%	40 618	73.91%	16 271	32.55%	33 711	67.45%
2007	14 460	26.14%	40 868	73.86%	16 521	32.75%	33 922	67.25%
2008	14 491	26.17%	40 887	73.83%	16 628	32.82%	34 037	67.18%
2009	14 493	25.81%	41 663	74.19%	16 738	32.53%	34 738	67.48%
2010	14 865	26.06%	42 180	73.94%	17 106	32.62%	35 331	67.38%
2011	15 244	26.63%	41 945	73.37%	17 111	32.68%	35 244	67.32%
2012	15 945	27.88%	41 256	72.12%	17 582	33.60%	34 752	66.40%
2013	15 924	28.36%	40 226	71.64%	17 751	34.40%	33 850	65.60%
2014	16 003	28.75%	39 654	71.25%	18 032	35.13%	33 299	64.87%
2015	16 712	29.71%	39 532	70.29%	18 571	35.91%	33 147	66.33%
2016	16 975	30.38%	38 907	69.62%	18 929	36.80%	32 513	63.20%
2017	18 243	33.49%	36 229	66.51%	20 110	40.00%	30 164	60.00%

### Out-migration rates: 2005–2017

**Fig. 1:**
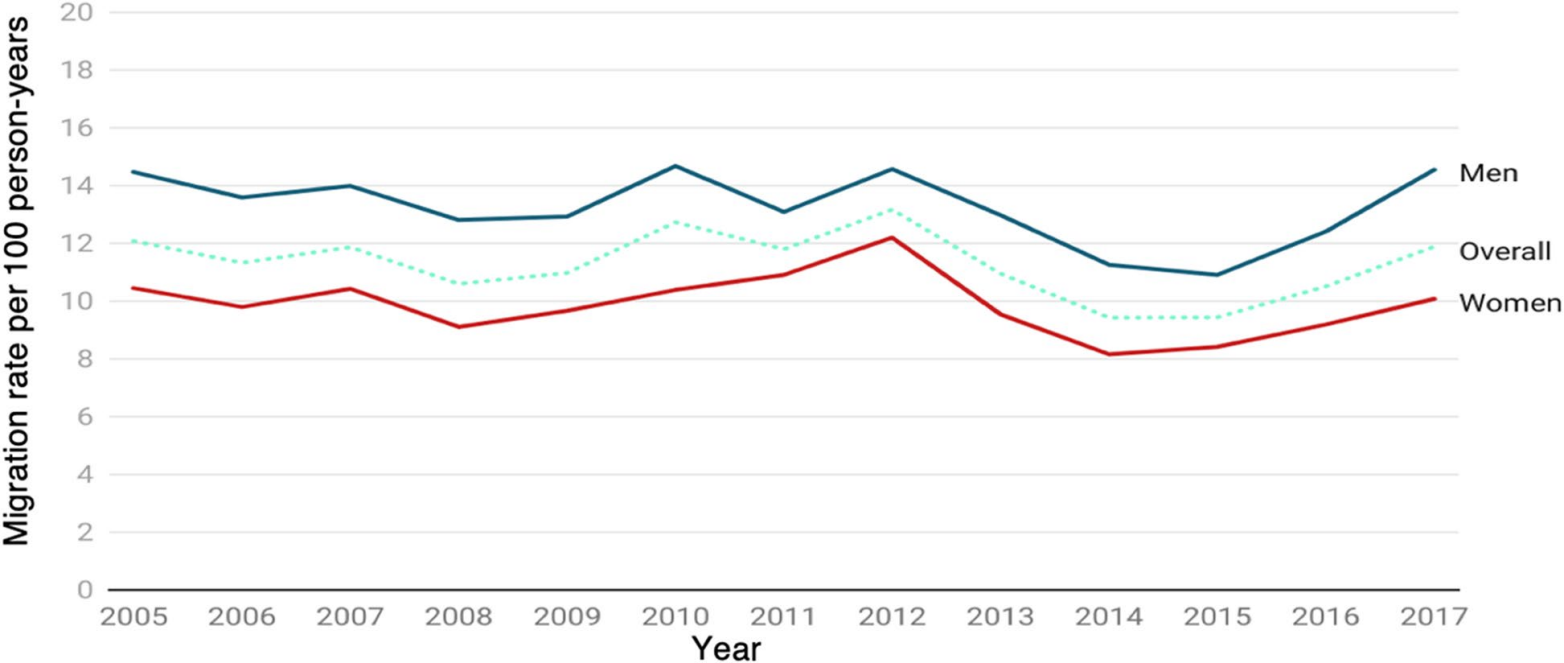
Trends of out-migration rates from a rural area in South Africa: 2005–2017

Crude annual migration rates for the observation period are presented in Table 2 and Fig. 1, ranging from 9.37 to 13.17 per 100 PY for both males and females. Overall, migration rates were high and fluctuating from 2005 to peak until 2009, after which they rose steadily to secondary peaks in 2010 and 2012, beyond which they declined.

**Table 2:** Trends of migration rates by year (2005–2017)

Year	Number of out-migration events	Person-years	Migration rate (per 100 person-years)	95% CI
2005	3908	32,340	12.08	11.71 – 12.47
2006	3695	32,604	11.33	10.97 – 11.70
2007	3884	32,733	11.87	11.50 – 12.24
2008	3510	33,120	10.60	10.25 –10.95
2009	3709	33,771	10.98	10.63 – 11.34
2010	4362	34,262	12.73	12.36 – 13.11
2011	4055	34,355	11.80	11.44 – 12.17
2012	4461	33,860	13.17	12.79 – 13.56
2013	3642	33,271	10.95	10.60 – 11.31
2014	3113	33,003	9.43	9.11 – 9.77
2015	3088	32,705	9.44	9.11 – 9.78
2016	3311	31,501	10.51	10.16 – 10.87
2017	3034	25,510	11.89	11.48 – 12.32

Highlighting other important data, the sex-specific trends of migration rates showed slightly bimodal patterns (Fig. 1), with the migration rates in women and men being similar across the years under observation. While migration was slightly lower in women than men, number of events for women per year increased by 0.53 and 0.25 events for men, particularly between 2008 and 2012.

### Incidence of out-migration: socio-demographic and health factors

Over the duration of the study (2005–2017), the crude migration rate was 11.29 events (95% confidence interval CI 11.19—11.39) per 100 person-years (47 772) events in 423 038 person-years of follow-up. Stratified by sex, the migration incidence rate was 9.96 events (95% CI 9.84 – 10.09) per 100 person-years (24 990 migration events in 250 833 person-years of follow-up) in women, while the rate was 13.23 events (95% CI 13.06—13.40) per 100 person-years (22 782 migration events in 172 205 person-years of follow-up) among men (Table 3). The incidence rate for migration among HIV negative individuals was 4.14 events (95% CI 4.04 – 4.25) per 100 person-years, while PLHIV had 8.86 events per (95% CI 8.62 – 9.12) per 100 person-years, and there were 17.15 events (95% CI 16.98 – 17.33) among those with unknown HIV-status.

**Table 3:** Incidence of out-migration among resident adults from a rural South African cohort study (*N* = 69 604)

		No. of events	Person-years at risk	Incidence rate (per 100 person-yrs)	95% CI
Age category:	15-19y	11 676	89 098	13.10	12.87 – 13.34
	20-24y	15 810	69 852	22.63	22.28 – 22.99
	25-29y	8 874	50 003	17.75	17.38 – 18.12
	30-34y	4 374	37 177	11.77	11.42 – 12.12
	35-39y	2 404	29 039	8.28	7.95 – 8.62
	40y +	4 634	147 869	3.13	3.04 – 3.23
Sex:	Male	22 782	172 205	13.23	13.06 – 13.40
	Female	24 990	250 833	9.96	9.84 – 10.09
Educational attainment:	None	5 264	77 896	6.76	6.58 – 6.94
	Primary < = 7y	2 932	50 814	5.77	5.56 – 5.98
	Secondary 8-12y	30 005	206 080	14.56	14.40 – 14.73
	Tertiary > 12y	2 025	19 776	10.24	9.80 – 10.70
	Unknown education status	7 546	68 472	11.02	10.77 – 11.27
Marital status:	Single	36 991	257 152	14.38	14.24 – 14.53
	Married	1 668	64 432	2.59	2.47 – 2.72
	Divorced/Separated	624	39 871	1.57	1.45—1.69
	Unknown marital status	8489	61 672	13.76	13.47 – 14.06
HIV status:	HIV-	6 446	155 589	4.14	4.04 – 4.25
	HIV +	4 865	54 879	8.86	8.62 – 9.12
	HIV status unknown	36 461	212 570	17.15	16.98 – 17.33

*ART* (Antiretroviral Therapy) *HIV*- (HIV Negative) *HIV* + (HIV positive) *CI* (Confidence Intervals)

**Fig. 2:**
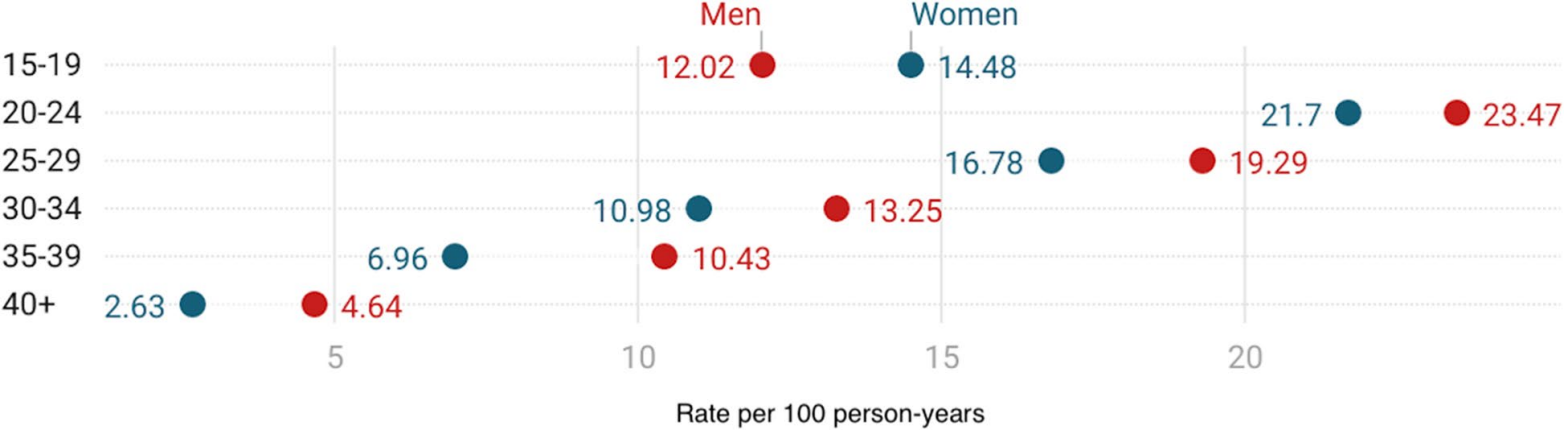
Migration rates by age and sex for the 2005–2017

The age-sex-specific distribution of migration rates was similar in both women and men across the years under observation, with the highest incidence rates among young adults aged 20–24 years (Fig. 2), which declined overall at ages > 25 years.

### Survival data description and summary

Over the course of the study (2005–2017), a total of 47 802 out-migration events accrued in 423 038 person-years of follow-up for 69 604 participants in the cohort. Nearly 69% of all study participants had at least one migration event over 525 397 observations. The mean follow-up time was 4.3 years (SEM 0.01) per individual, while the median number of migration events per person was four (IQR 2–7), ranging from 1 to 14. The median time to migration by HIV status was 5.9 years (95% CI 5.8 – 6.1) in PLHIV and 3.1 years (95% CI 3.0 – 3.1) in those with unknown HIV status.

Table 4 shows the Andersen-Gill Model regression results for predictors of external migration events, with increased potential being associated with being aged 20–24 years (adjusted hazard ratio [aHR] = 3.11, 95% CI 2.99 – 3.24) and 25–29 years (aHR = 2.30, 95% CI 2.20 – 2.40) compared to 40 years and older. Individuals who were single had a two times higher hazard of external migration than those who were married (aHR = 2.28, 95% CI 2.15 – 2.41). Community ART coverage was among the most important factors significantly associated with migration in both the unadjusted and adjusted models. The risk of migration was 59% higher per every 1% increment in community ART coverage (HR = 1.59, 95% CI 1.49 – 1.69) and 16% lower per every 1% increment in community ART coverage (aHR = 0.84, 95% CI 0.79–0.91).

**Table 4:** Determinants of out-migration in a rural South African cohort study (*N* = 69 604)

	Category	HR	SE	95% CI	aHR	SE	95% CI
Sex: [Female]	Male	1.24***	0.01	1.21 – 1.26	0.97***	0.01	0.95 – 0.99
Age category: [≥ 40y]	15-19y	3.29***	0.06	3.17 – 3.42	2.05***	0.05	1.96—2.14
	20-24y	5.19***	0.11	5.71 – 6.13	3.11***	0.06	2.99 – 3.24
	25-29y	4.56***	0.09	4.39 – 4.74	2.30***	0.05	2.20 – 2.40
	30-34y	3.11***	0.07	2.98 – 3.25	1.63***	0.04	1.56 – 1.72
	35-39y	2.32***	0.06	2.20 – 2.43	1.38***	0.04	1.31 – 1.45
Educational attainment: [Primary]	None	1.13***	0.03	1.08 – 1.19	0.90***	0.02	0.85 – 0.94
	Secondary	2.31***	0.05	2.21 – 2.40	1.26***	0.03	1.21 – 1.31
	Tertiary	1.76***	0.05	1.66 – 1.87	1.26***	0.04	1.19 – 1.34
	Unknown education status	1.63***	0.04	1.56 – 1.72	0.91***	0.02	0.87 – 0.96
Marital status: [Currently married]	Single	4.66***	0.13	4.42 – 4.92	2.28***	0.07	2.15 – 2.41
	Separated/Divorced	0.64***	0.03	0.58 – 0.70	0.86***	0.04	0.78 – 0.95
	Unknown marital status	4.45***	0.13	4.20 – 4.71	2.58***	0.09	2.42 – 2.76
HIV status: [HIV-]	HIV +	2.58***	0.05	2.48 – 2.68	2.44***	0.05	2.35 – 2.54
	Unknown HIV status	4.65***	0.07	4.53 – 4.79	4.15***	0.06	4.03 – 4.26
ART coverage	ART %	1.59***	0.06	1.49 – 1.69	0.84***	0.03	0.79 – 0.91

*aHR* (Adjusted Hazard Ratio), *SE* (Standard error), *ART* (Antiretroviral Therapy), *CI* (Confidence intervals), *HR* (Hazard ratio), *HIV*- (HIV Negative) *HIV* + (HIV positive). Final models were adjusted for sex, age, educational attainment, marital status, HIV status and community ART coverage

^***^p < 0.05 [Reference category in brackets]

Table 5 shows the results for the analyses stratified by gender, with out-migration being three times higher among women aged 20–24 years (aHR = 3.37, 95% CI 3.19 – 3.57) compared to those aged ≥ 40 years, while for men, those aged 20–24 years were 2.9 times more likely to migrate compared to those aged ≥ 40 years. Nearly all covariates, including sex, age, education and marital status, were significantly associated with migration in the unadjusted and adjusted models for both women and men. HIV and ART status were strongly associated with the migration incidence among both women and men. Women who were HIV positive were 2.4 times more likely to migrate, compared to HIV-negative individuals in the unadjusted model, and such hazard remaining significant in the multivariate model (aHR = 2.44, 95% CI 1.58–1.77). Similarly, the hazard of migration was 2.5-times higher (aHR = 2.54,95% CI 2.36–2.73) among HIV positive than HIV negative women. In separate models for women (aHR = 4.18, 95% CI 4.03 – 4.34) and men (aHR = 2.44, 95% CI 3.89 – 4.23), migration risk increased by more than 4-times for those who were HIV positive compared to those who were negative. Community ART coverage was among the most important factors significantly associated with migration in both the unadjusted and adjusted models. Every 1% increase in community ART coverage lowered migration among women (aHR = 0.91, 95% CI: 0.83 – 0.99) and men (aHR = 0.73, 95% CI 0.66 – 0.82).

**Table 5:** Determinants of out-migration in a rural area in South Africa by gender (*N* = 69 604)

Category		HR	95% CI	aHR	95% CI	HR	95% CI	aHR	95% CI
		Female				Male			
		*N* = 38 372				*N* = 30 612			
Age category: [≥ 40y]	15-19y	4.32***	4.11 – 4.55	2.32***	2.18 – 2.47	2.18***	2.06 – 2.31	1.80***	1.68 – 1.92
	20-24y	6.81***	6.49 – 7.15	3.37***	3.19 – 3.57	4.50***	4.27 -4.75	2.86***	2.69 – 3.04
	25-29y	5.12***	4.86 – 5.39	2.43***	2.29 – 2.58	3.61***	3.41 – 3.83	2.17***	2.04 – 2.32
	30-34y	3.49***	3.29 – 3.70	1.76***	1.65 – 1.88	2.48***	2.32 – 2.64	1.52***	1.41 – 1.63
	35-39y	2.38***	2.22 – 2.55	1.39***	1.29 – 1.49	2.07***	1.92 – 2.22	1.37***	1.27 – 1.48
Education status: [Primary]	None	1.31***	1.22 – 1.40	0.92	0.86 – 0.98	0.94***	0.87 – 1.00	0.85***	0.80 – 0.91
	Secondary	2.72***	2.57 – 2.88	1.26***	1.19 – 1.34	1.82***	1.72 – 1.92	1.22***	1.16 – 1.29
	Tertiary	2.11***	1.95 – 2.29	1.29***	1.19 – 1.40	1.40***	1.28 – 1.53	1.20***	1.09 – 1.31
	Unknown Educational status	1.87***	1.75 – 2.01	0.88***	0.82 – 0.94	1.35**	1.27 – 1.44	0.93**	0.87 – 0.99
Marital status: [Currently Married]	Single	5.18***	4.81 –5.57	2.49***	2.30 – 2.69	3.97***	3.68 – 4.28	2.04***	1.87 – 2.22
	Separated/Divorced	0.72***	0.64 – 0.80	0.96	0.86 – 1.08	0.97	0.77 – 1.21	1.08	0.86 – 1.35
	Unknown marital status	5.98***	5.53 – 6.47	3.15***	2.88 – 3.45	3.03***	2.78 – 3.29	2.00***	1.82 – 2.21
HIV status: [HIV-]	HIV +	2.79***	2.66 – 2.93	2.44***	2.33 – 2.57	2.26***	2.11 – 2.42	2.54***	2.36 – 2.73
	Unknown HIV status	4.87***	4.68 – 5.06	4.18***	4.03 – 4.34	4.23***	4.06 – 4.41	4.05***	3.89 – 4.23
ART coverage	ART%	2.64***	2.44 – 2.87	0.91***	0.83–0.99	0.97	0.88 – 1.07	0.73***	0.66 – 0.82

*aHR* (Adjusted Hazard Ratio), *SE* (Standard error) *ART* (Antiretroviral Therapy), Confidence intervals (CI), *HR* (Hazard ratio), *HIV-* (HIV Negative) *HIV* + (HIV positive). Final models were adjusted for age, educational attainment, marital status, HIV status and community ART coverage

^***^p < 0.05 [Reference category in brackets]

Based on the results from the complete case analysis (i.e. limiting analysis to non-missing data of variables relevant variables), the effects of socio-demographic characteristics (age and education status) on the migration risk profiles were similar to the estimates with the models in Tables 4 and 5 covariate information (Tables S2, S3). Similarly, in other sensitivity analyses, migration hazard by unknown HIV status and individuals with HIV positive and HIV negative status were comparable with the main models. The aHR for ages 20–24 and 25–29 compared to 40 + were 1.86; 3.33 for those HIV positive, 1.91; 2.88 for those with unknown HIV status, and 3.52; 4.78 for those HIV negative (S4-S6).

## Discussion

In a rural South African community, between 2005 and 2017, we found that out-migration rates remained consistently high, with variance by sex. The risk of migration was three times higher among young adults (20–24 years) compared to older adults ≥ 40 years, and two times higher among unmarried compared to married women and men. Notably, PLHIV had a two-fold increased hazard of migration when compared to HIV-negative men and women. However, increased community ART coverage lowered migration risk by 9% in women and 27% in men. These findings reflect a necessary focus on the context of mobility in SSA, a reality often overlooked in the development HIV treatment and prevention programs.

The socio-demographic profiles of migrating in our study were in keeping with the commonly held image of migrants from prior research in SSA. The high proportion of young women and men of working age with secondary education among migrants may reflect the persistence of historical labour-related mobility from rural to urban settings to seek greater employment opportunities [40–46]. Notably, this near-convergence of migration by sex, if conducted for remunerative purposes may help improve the de-facto economic standing of female-headed households, currently poorer than male-headed households in South Africa [47]. Moreover, transition into early adulthood is an important life-course marker that is synonymous with establishing independent adult status, and often requires out-migration [48]. Our results show that adolescent girls migrated more than boys in our study possibly confirming that women adopt adult earlier roles compared men [49]. Taken together, this evidence highlights the complex interplay of motivations for young people to move within contexts of high HIV-risk in pursuit of economic opportunity [48, 50].

Beyond individual characteristics, meso- and macro-level factors are often important considerations for change of residence for PLHIV. There is evidence that in the context of limited treatment, an HIV diagnosis increases external migration as individuals move either to avoid social stigma from their communities or seek better palliative care elsewhere [19, 51, 52]. Previously, out-migrants from the study area (likely to urban areas with specialized HIV related services) had a lower risk of dying from AIDS compared to residents [19], signaling the association of movements outside poor communities with better health outcomes for PLHIV. Fortunately, in 2014, the UTT interventions and accompanying 95–95-95 targets in KwaZulu-Natal Province and elsewhere were implemented, where universal roll-out of HIV services and antiretroviral therapy commenced for consenting adults and PLHIV, an estimated 70% in KZN are currently accessing therapy [53]. The wider coverage of ART in this community lowered migration, after adjusting for the key socio-demographic-clinical variables, possibly demonstrating alleviated problems related to the improved geographic accessibility of HIV services.

Our findings highlight the need to intensify the engagement and retention of migrants in effective combinations of HIV prevention and care programs in the era of ART. That ART coverage reduced migration may be illustrative of successful engagement to care in local clinics among those living with HIV, while increased mobility for PLHIV has implications for high onward transmission. We therefore echo others ‘recommendations that mobile populations may benefit from novel models of differentiated care that simplify and adapt HIV services across the cascade for PLHIV and to decongest the health system [33, 54]. These models include patient-led community adherence groups, sexual networks [55, 56], healthcare worker-managed groups, fast-track or multi-month drug scripting, mobile outreach, interventions optimized to seasonal migration patterns [57] and community drug distribution points (e.g., pharmacy-based refills). Importantly, these models can be facilitated by an acute awareness of the needs of mobile women and men living with HIV, holding the promise for engaging and retaining these populations who struggle to fit their needs to the requirements of community/clinic-based HIV care systems. Beyond social and structural interventions, mobile populations may benefit from improved therapeutic technologies, such as long-acting ART regimens, which should be accompanied by extending their coverage into communities and key migration destinations, including transit hubs [58]. Recognizing that adolescent girls migrate more (and are high HIV incidence cohort) approaches sensitive to age and gender, such as self-testing kits [59–61] and pre-exposure prophylaxis kits [

The strengths of our study include the follow-up of more than 69 604 men and women participating in one of the world’s largest and longest running demographic and HIV cohorts, which enabled us to capture long-term trends in adult migration (i.e., 2005–2017) over a period when ART was scaled-up in South Africa. In addition, we analysed comprehensive data on the migration history of both male and female participants for close to 12-years, which helped us to not only measure levels and trends of migration, but also the effect of long-term time-varying covariates. Linking migrant characteristics with positive selection for incident migration will help disentangle the mechanisms behind the association between migration and HIV risk as the larger epidemiologic context changes in sSA. This effort leverages our previous work demonstrating that the risk of HIV acquisition conditional to migration increased by 70% (aOR = 1.69, 95% CI: 2.33 – 2.14; *I*^2^ = 35.0%) between 2000 – 2017 [11]. We also used Andersen-Gill models to address frequent and recurrent migratory events per individual in the study area, adjusting for time-varying demographic and health covariates.

This study is not without limitations, with some participants not always being available at each successive round for HIV data collection being a challenge in the AHRI surveillance area, as in large and long-running studies elsewhere [30]. The lack of HIV ascertainment issue was noted by Vandormael and colleagues, who showed that over a 12-year period in the study area between 2005 and 2017, it is unlikely that HIV incidence rates were significantly affected by potential selection bias due to participants missing scheduled HIV tests [4]. We posit that mobility often rendered participants temporarily unavailable for HIV testing during scheduled visits in the study site, contributing to the observed missingness [8]. Furthermore, we examined out-migration from the region and not in-migration, and thus did not explore concomitant mobility dynamics. However, given our large sample size and consistently high response rates in the AHRI household data collection surveys (> 99%), we believe that our results indicate a fairly balanced picture of the socio-demographic and health factors of migration among adults in rural KZN. We also show that the results from a complete case analysis (i.e., limiting the analysis to non-missing data of variables relevant variables), their socio-demographic characteristics (age and education status) and migration risk profiles were largely consistent, with estimates of missing covariate information (Supplementary Tables S2, S3). For additional sensitivity analyses, we fitted models for the hazard of migration by HIV status categories (i.e., variable accounting for the largest missingness), comparing the results of unknown HIV status with those HIV positive and negative. The results were comparable overall, with similarities in the aHR for ages 20–24 and 25–29 compared to 40 + , i.e., 1.86; 3.33 for those HIV positive, 1.91; 2.88 for those with unknown HIV status, and 3.52 and 4.78 for those HIV negative. The magnitude and direction of effect of education status on migration was similar across the various HIV scenarios, particularly for those with secondary and tertiary level education, who had a higher aHR than those with primary education (Table S4, S5 and S6).

## Conclusions

Our study provides evidence of the diverse migration characteristics of mobile men and women from a hyper-endemic community in South Africa. While the results highlight that migration remains a male domain, younger adults (i.e., both women and men), those unmarried and HIV positive were more likely to migrate, indicating the changing profile of migrants during an era of ART in an hyper-endemic community. Differentiated prevention and care interventions customized to the unique HIV risks and needs of mobile women and men are needed to reduce the risk of HIV acquisition and onward transmission among this highly vulnerable group.

## Supplementary Information

**Additional file 1:** **Table S1.** Reasons for migration from rural area in South Africa 2005-2017. **Table S2.** Determinants of out-migration in a rural South African cohort study based on complete case analyses (*N*=41 136). **Table S3.** Determinants of out-migration in a rural area in South Africa by gender (*N*=39 267). **Table S4. **Sensitivity analyses - Determinants of out-migration among those HIV negative in a rural South African cohort study (*N*=31 346). **Table S5.** Determinants of out-migration among those HIV positive in a rural South African cohort study (*N*=10 754). **Table S6.** Determinants of out-migration among those with unknown HIV status in a rural South African cohort study (*N*=60 214).

## Data Availability

Data used in this analysis are available to the public domain upon request from the AHRI repository https://data.africacentre.ac.za/index.php/auth/login/?destination.

## Abbreviations

HIV: Human Immune Virus
ART: Antiretroviral Therapy
AIDS: Acquired Immune Deficiency Syndrome
PLHIV: People Living with HIV
SSA: Sub-Saharan Africa
UTT: Universal Test and Treat
AHRI: Africa Health Research Institute
KZN: KwaZulu-Natal
PHC: Primary Health Care
CAB: Community Advisory Board
BREC: Biomedical Research Ethics Committee
PY: Person-Years
CI: Confidence Interval
SEM: Standard Error of the Mean
IQR: Interquartile Range
HR: Hazard Ratio
AHR: Adjusted Hazard Ratio
