# Supplementary material for: Predictors of migration in an HIV hyper-endemic rural South African community: evidence from a population-based cohort (2005–2017)
Source: BMC Public Health. 2022 Jun 7;22:1141. doi: 10.1186/s12889-022-13526-w (PMC9175358; doi:10.1186/s12889-022-13526-w)
Supplement: Supplementary file 1 — Additional file 1: Table S1. Reasons for migration from rural area in South Africa 2005-2017. Table S2. Determinants of out-migration in a rural South African cohort study based on complete case analyses (N=41 136). Table S3. Determinants of out-migration in a rural area in South Africa by gender (N=39 267). Table S4. Sensitivity analyses - Determinants of out-migration among those HIV negative in a rural South African cohort study (N=31 346). Table S5. Determinants of out-migration among those HIV positive in a rural South African cohort study (N=10 754). Table S6. Determinants of out-migration among those with unknown HIV status in a rural South African cohort study (N=60 214). [file 12889_2022_13526_MOESM1_ESM.docx]

**Supplementary Tables**

**Table S1: Reasons for migration from rural area in South Africa 2005-2017**

| Reasons for migration | Migrations events (49 198) | |  |
| --- | --- | --- | --- |
|  | (*N*) | (%) | (95% CI) |
| Accommodation | 20 913 | 28.54 | 28.15 – 28.95 |
| Education | 15 851 | 21.5 | 21.13 – 21.86 |
| Employment | 27 832 | 36.84 | 36.41– 37.27 |
| Care for someone | 4 523 | 9.15 | 8.90 – 9.41 |
| Violence and crime | 436 | 0.86 | 0.78 – 0.95 |
| Marital | 755 | 1.47 | 1.36 – 1.57 |
| Health | 824 | 1.63 | 1.52 – 1.75 |

Note: Based on non-missing migration event data for reason for change of residency at time of observation. Abbreviation: (CI) Confidence Interval

**Table S2: Determinants of out-migration in a rural South African cohort study based on complete case analyses (N=41 136)**

|  | Category | HR | SE | 95% CI | aHR | SE | 95% CI |
| --- | --- | --- | --- | --- | --- | --- | --- |
|  | Male | 1.24* | 0.01 | 1.21 – 1.26 | 1.02 | 0.01 | 0.99 – 1.05 |
| Age category: [≥40y] | 15-19y | 3.29* | 0.06 | 3.17 – 3.42 | 3.10* | 0.12 | 2.88 – 3.34 |
|  | 20-24y | 5.91* | 0.11 | 5.71 – 6.13 | 3.87* | 0.13 | 3.63 – 4.13 |
|  | 25-29y | 4.56* | 0.09 | 4.39 – 4.74 | 2.67* | 0.09 | 2.50 – 2.85 |
|  | 30-34y | 3.11* | 0.07 | 2.98 – 3.25 | 1.73* | 0.06 | 1.61 – 1.87 |
|  | 35-39y | 2.32* | 0.06 | 2.20 – 2.43 | 1.46* | 0.04 | 1.34 – 1.58 |
| Educational attainment: [Primary] | None | 1.12* | 0.03 | 1.08 – 1.17 | 0.93* | 0.02 | 0.87 – 1.00 |
|  | Secondary | 2.28* | 0.05 | 2.19 – 2.38 | 1.37* | 0.04 | 1.30 – 1.45 |
|  | Tertiary | 1.75* | 0.05 | 1.65 – 1.86 | 1.51* | 0.07 | 1.19 – 1.34 |
| Marital status: [Married] | Single | 4.66* | 0.13 | 4.42 – 4.92 | 2.03* | 0.09 | 1.86 – 2.21 |
|  | Separated/Divorced | 0.64* | 0.03 | 0.58 – 0.70 | 0.86* | 0.06 | 0.75 – 0.99 |
| HIV status: [HIV-] | HIV+ | 2.50* | 0.05 | 2.40 – 2.60 | 2.49* | 0.06 | 2.38 – 2.60 |
| ART coverage | ART % | 1.80* | 0.06 | 1.68 – 1.91 | 0.57* | 0.03 | 0.52 – 0.66 |

***p<0.05 [Reference category in brackets]. Abbreviations: aHR (Adjusted Hazard Ratio), SE (Standard error), ART (Antiretroviral Therapy), Confidence intervals (CI), Hazard ratio (HR), HIV- (HIV Negative) HIV+ (HIV positive). Final models were adjusted for, sex, age, educational attainment, marital status, HIV status and community ART coverage.

**Table S3: Determinants of out-migration in a rural area in South Africa by gender (N=39 267)**

|  | Category | HR | 95% CI | aHR | 95% CI | | HR | | 95% CI | | aHR | 95% CI | |  |
| --- | --- | --- | --- | --- | --- | --- | --- | --- | --- | --- | --- | --- | --- | --- |
|  |  |  | Female | | |  | |  | | Male | | |  | |
|  |  |  | N=23 391 | | |  | |  | | N=15 876 | | |  | |
| Age category:[≥40y] | 15-19y | 4.32* | 4.11 – 4.55 | 4.62* | 4.20 – 5.09 | | 2.18* | | 2.06 – 2.31 | | 2.97* | 2.65 – 3.32 | |  |
|  | 20-24y | 6.81* | 6.49 – 7.15 | 4.77* | 4.37 – 5.21 | | 4.50* | | 4.27 -4.75 | | 3.84* | 3.46 – 4.26 | |  |
|  | 25-29y | 5.12* | 4.86 – 5.39 | 3.21* | 2.93 – 3.52 | | 3.61* | | 3.41 – 3.83 | | 2.70* | 2.43 – 3.01 | |  |
|  | 30-34y | 3.49* | 3.29 – 3.70 | 2.13* | 1.93 – 2.35 | | 2.48* | | 2.32 – 2.64 | | 1.67* | 1.49 – 1.88 | |  |
|  | 35-39y | 2.38* | 2.22 – 2.55 | 1.67* | 1.50 – 1.87 | | 2.07* | | 1.92 – 2.22 | | 1.48* | 1.29 – 1.69 | |  |
| Education status:[Primary] | None | 1.28* | 1.20 – 1.37 | 0.98 | 0.89 – 1.08 | | 0.93* | | 0.87 – 1.00 | | 0.95 | 0.85 – 1.05 | |  |
|  | Secondary | 2.67* | 2.52 – 2.83 | 1.34* | 1.23 – 1.46 | | 1.81* | | 1.71 – 1.91 | | 1.37* | 1.25 – 1.49 | |  |
|  | Tertiary | 2.09* | 1.93 – 2.27 | 1.49* | 1.33 – 1.67 | | 1.39* | | 1.27 – 1.52 | | 1.46* | 1.28 – 1.67 | |  |
| Marital status:[Married] | Single | 4.21* | 3.95 – 4.48 | 1.75* | 1.58 – 1.94 | | 3.42* | | 3.19 – 3.66 | | 1.51* | 1.32 – 1.72 | |  |
|  | Separated/Divorced | 0.72* | 0.65 – 0.79 | 1.00 | 0.87 – 1.16 | | 1.00 | | 0.82 – 1.22 | | 1.10 | 0.80 – 1.51 | |  |
| HIV status:[HIV-] | HIV+ | 2.69* | 2.56 – 2.83 | 2.35* | 2.22 – 2.48 | | 2.23* | | 2.08 – 2.39 | | 2.61* | 2.40 – 2.83 | |  |
| ART coverage | ART% | 2.85* | 2.61 – 3.11 | 0.99 | 0.86 – 1.16 | | 0.97 | | 0.90 – 1.08 | | 0.57* | 0.48 – 0.68 | |  |

***p<0.05 [Reference category in brackets]. Abbreviations: aHR (Adjusted Hazard Ratio), SE (Standard error), ART (Antiretroviral Therapy), Confidence intervals (CI), Hazard ratio (HR), HIV- (HIV Negative) HIV+ (HIV positive). Final models were adjusted for, age, educational attainment, marital status, HIV status and community ART coverage.

**Table S4: Sensitivity analyses - Determinants of out-migration among those HIV negative in a rural South African cohort study (N=31 346)**

|  | Category | HR | SE | 95% CI | aHR | SE | 95% CI |
| --- | --- | --- | --- | --- | --- | --- | --- |
|  | Male | 1.19* | 0.03 | 1.13 – 1.25 | 0.97* | 0.02 | 0.92 – 1.02 |
| Age category: [≥40y] | 15-19y | 4.04* | 0.21 | 3.65 – 4.48 | 3.52* | 0.23 | 3.09 – 4.01 |
|  | 20-24y | 9.10* | 0.45 | 8.26 – 10.03 | 4.78* | 0.31 | 4.21 – 5.41 |
|  | 25-29y | 7.01* | 0.40 | 6.27 – 7.84 | 3.71* | 0.26 | 3.24 – 4.25 |
|  | 30-34y | 3.87* | 0.29 | 3.34 – 4.49 | 2.23* | 0.19 | 1.89 – 2.64 |
|  | 35-39y | 3.01* | 0.25 | 2.55 – 2.55 | 2.02* | 0.18 | 1.70 – 2.40 |
| Educational attainment: [Primary] | None | 0.67* | 0.04 | 0.59 – 0.76 | 0.80* | 0.05 | 0.70 – 0.91 |
|  | Secondary | 2.92* | 0.15 | 2.65 – 3.22 | 1.48* | 0.08 | 1.34 – 1.64 |
|  | Tertiary | 1.78* | 0.17 | 1.47 – 2.15 | 1.41* | 0.14 | 1.17 – 1.71 |
|  | Unknown education status | 1.91* | 0.12 | 1.69 – 2.16 | 1.28* | 0.08 | 1.14 – 1.45 |
| Marital status: [Married] | Single | 5.33* | 0.33 | 4.72 – 6.04 | 2.01* | 0.15 | 1.74 – 2.32 |
|  | Separated/Divorced | 0.64* | 0.07 | 0.52 – 0.80 | 0.87* | 0.10 | 0.70 – 1.09 |
|  | Unknown marital status | 1.33* | 0.11 | 1.33 – 1.55 | 0.80* | 0.08 | 0.67 – 0.73 |
| ART coverage | ART % | 0.17* | 0.02 | 0.15 – 0.21 | 0.21* | 0.02 | 0.17 – 0.26 |

***p<0.05 [Reference category in brackets]. Abbreviations: aHR (Adjusted Hazard Ratio), SE (Standard error), ART (Antiretroviral Therapy), Confidence intervals (CI), Hazard ratio (HR), HIV- (HIV Negative) HIV+ (HIV positive). Final models were adjusted for sex, age, educational attainment, marital status, HIV status and community ART coverage.

**Table S5: Determinants of out-migration among those HIV positive in a rural South African cohort study (N=10 754)**

|  | Category | HR | SE | 95% CI | aHR | SE | 95% CI |
| --- | --- | --- | --- | --- | --- | --- | --- |
|  | Male | 1.00 | 0.03 | 0.94 – 1.07 | 1.06 | 0.04 | 0.99 – 1.14 |
| Age category: [≥40y] | 15-19y | 3.04* | 0.24 | 2.61 – 3.54 | 1.86* | 0.17 | 1.56 - 2.22 |
|  | 20-24y | 3.99* | 0.19 | 3.62 – 4.39 | 3.33* | 0.19 | 2.99 – 3.70 |
|  | 25-29y | 3.08* | 0.15 | 2.80 – 3.39 | 2.54* | 0.13 | 2.29 – 2.81 |
|  | 30-34y | 2.21* | 0.11 | 2.00 – 2.45 | 1.84* | 0.10 | 1.65 – 2.04 |
|  | 35-39y | 1.69* | 0.10 | 1.51 – 1.89 | 1.45* | 0.09 | 1.29 – 1.63 |
| Educational attainment: [Primary] | None | 1.28* | 0.10 | 1.09 – 1.50 | 1.01 | 0.08 | 0.86 – 1.19 |
|  | Secondary | 1.96* | 0.12 | 1.74 – 2.21 | 1.28* | 0.08 | 1.14 – 1.45 |
|  | Tertiary | 1.84* | 0.16 | 1.54 – 2.19 | 1.50* | 0.13 | 1.26 – 1.78 |
|  | Unknown education status | 2.27* | 0.17 | 1.95 – 2.64 | 1.45* | 0.11 | 1.24 – 1.68 |
| Marital status: [Married] | Single | 2.86* | 0.23 | 2.44 – 3.35 | 1.79* | 0.15 | 1.52 – 2.11 |
|  | Separated/Divorced | 0.79* | 0.11 | 0.61 – 1.03 | 0.96 | 0.13 | 0.74 – 1.26 |
|  | Unknown marital status | 7.83* | 0.70 | 6.58 – 9.33 | 5.66* | 0.53 | 4.71 – 6.80 |
| ART coverage | ART % | 1.77* | 0.24 | 1.36 – 2.30 | 1.40* | 0.20 | 1.06 – 1.85 |

***p<0.05 [Reference category in brackets]. Abbreviations: aHR (Adjusted Hazard Ratio), SE (Standard error), ART (Antiretroviral Therapy), Confidence intervals (CI), Hazard ratio (HR), HIV- (HIV Negative) HIV+ (HIV positive). Final models were adjusted for, sex, age, educational attainment, marital status, HIV status and community ART coverage.

**Table S6: Determinants of out-migration among those with unknown HIV status in a rural South African cohort study (N=60 214)**

|  | Category | HR | SE | 95% CI | aHR | SE | 95% CI |
| --- | --- | --- | --- | --- | --- | --- | --- |
|  | Male | 1.06* | 0.01 | 1.04 – 1.09 | 0.96* | 0.01 | 0.93 – 0.98 |
| Age category: [≥40y] | 15-19y | 3.08* | 0.07 | 2.94 – 3.22 | 1.91* | 0.05 | 1.82 - 2.01 |
|  | 20-24y | 4.44* | 0.10 | 4.26 – 4.63 | 2.88* | 0.10 | 2.75 – 3.02 |
|  | 25-29y | 3.28* | 0.08 | 3.14 – 3.44 | 2.14* | 0.05 | 2.03 – 2.25 |
|  | 30-34y | 2.22* | 0.06 | 2.11 – 2.34 | 1.54* | 0.04 | 1.46 – 1.63 |
|  | 35-39y | 1.70* | 0.05 | 1.59 – 1.79 | 1.29* | 0.04 | 1.22 – 1.37 |
| Educational attainment: [Primary] | None | 1.10* | 0.03 | 1.04 – 1.16 | 0.91* | 0.03 | 0.86 – 0.96 |
|  | Secondary | 1.67* | 0.04 | 1.60 – 1.76 | 1.19* | 0.03 | 1.13 – 1.24 |
|  | Tertiary | 1.06 | 0.04 | 0.99 – 1.14 | 1.16* | 0.04 | 1.08 – 1.24 |
|  | Unknown education status | 1.01 | 0.03 | 0.96 – 1.06 | 0.80* | 0.02 | 0.76 – 0.84 |
| Marital status: [Married] | Single | 4.06* | 0.13 | 3.82 – 4.32 | 2.32* | 0.08 | 2.17 – 2.48 |
|  | Separated/Divorced | 0.75* | 0.05 | 0.66 – 0.84 | 0.87* | 0.05 | 0.77 – 0.98 |
|  | Unknown marital status | 3.77* | 0.13 | 3.53 – 4.03 | 2.74* | 0.10 | 2.54 – 2.95 |
| ART coverage | ART % | 1.94* | 0.07 | 1.80 – 2.08 | 1.05* | 0.04 | 0.96 – 1.14 |

***p<0.05 [Reference category in brackets]. Abbreviations: aHR (Adjusted Hazard Ratio), SE (Standard error), ART (Antiretroviral Therapy), Confidence intervals (CI), Hazard ratio (HR), HIV- (HIV Negative) HIV+ (HIV positive). Final models were adjusted for age, educational attainment, marital status, HIV status and community ART coverage.
